# AXL degradation in combination with EGFR-TKI can delay and overcome acquired resistance in human non-small cell lung cancer cells

**DOI:** 10.1038/s41419-019-1601-6

**Published:** 2019-05-01

**Authors:** Donghwa Kim, Duc-Hiep Bach, Yan-Hua Fan, Thi-Thu-Trang Luu, Ji-Young Hong, Hyen Joo Park, Sang Kook Lee

**Affiliations:** College of Pharmacy, Natural Products Research Institute, Seoul National University, Seoul, 08826 Korea

**Keywords:** Non-small-cell lung cancer, Target validation

## Abstract

Acquired resistance to epidermal growth factor receptor-tyrosine kinase inhibitors (EGFR-TKIs) has been a major obstacle in the treatment of non-small cell lung cancer (NSCLC) patients. AXL has been reported to mediate EGFR-TKIs. Recently, third generation EGFR-TKI osimertinib has been approved and yet its acquired resistance mechanism is not clearly understood. We found that AXL is involved in both gefitinib and osimertinib resistance using in vitro and in vivo model. In addition, AXL overexpression was correlated with extended protein degradation rate. We demonstrate targeting AXL degradation is an alternative route to restore EGFR-TKIs sensitivity. We confirmed that the combination effect of YD, an AXL degrader, and EGFR-TKIs can delay or overcome EGFR-TKIs-driven resistance in EGFR-mutant NSCLC cells, xenograft tumors, and patient-derived xenograft (PDX) models. Therefore, combination of EGFR-TKI and AXL degrader is a potentially effective treatment strategy for overcoming and delaying acquired resistance in NSCLC.

## Introduction

Epidermal growth factor receptor (EGFR) mutation is one of the major driver oncogenes in non-small cell lung cancer (NSCLC) and most frequently found in Asian patients^1–3^. Although the first generation of EGFR tyrosine kinase inhibitors (TKIs), such as gefitinib and erlotinib, have led to improved prognoses for NSCLC patients, their long-term efficacy is questionable due to the emergence of acquired resistance within a year of treatment^4,5^. Recently, a third generation EGFR-TKIs osimertinib, a specific inhibitor of mutant EGFR, has been approved for clinical use. However, several resistance mechanisms were subsequently identified from patients’ samples including C797S and L718Q EGFR mutations, SCLC transformation, HER2 amplification, and MET amplification^6^. Although a number of agents have been suggested for development to target the L858R/T790M/C797S triple mutation of EGFR, alternative approaches to control resistance are needed and the use of drug combinations may benefit patients who do not respond to current treatment^7,8^.

AXL is a receptor tyrosine kinase that belongs to the TAM family which consists of three members: Tyro3, MERTK, and AXL^9^. Dysregulation of TAM signaling has been reported to be associated with cancer, chronic inflammation, and autoimmune disease^10^. Among three TAM member, AXL, both growth arrest-specific gene 6 (GAS6)-dependent and (GAS6)-independent, can promote many downstream signaling pathways and transcription factors regulating cell survival, growth, EMT, metastasis, and tumor microenvironment in cancer cells^11–13^. Recently, AXL has been reported to play a role in drug resistance mechanisms for many anti-cancer drugs, as well as in ionizing radiation therapy for multiple cancers^14–17^. AXL receptor kinase inhibitors have shown profound effects in overcoming the acquired resistance to EGFR-TKIs in mesenchymal cancer cells, but their anti-proliferative effects as a single agent are very limited^18^. Since AXL is considered as an attractive target to overcome the resistance to EGFR-TKIs, several AXL kinase inhibitors, antibody drug conjugates and decoy receptors are currently under investigation in clinical trials for cancer treatment^19–21^.

In this study, we determined that activation of the AXL in EGFR-TKIs resistant cells is associated with extended protein degradation of AXL. We further demonstrated the combining YD, an AXL degrader, and EGFR-TKI resulted in overcoming resistance in EGFR-TKIs resistant NSCLC cells also delaying the emergence of resistance in EGFR-TKI sensitive NSCLC cells using tumor xenograft, and PDX model.

## Materials and methods

### Chemicals and reagents

Gefitinib (CAS No. HY-50895) and Osimertinib (AZD-9291, CAS No. HY-15772) were purchased from MedChemExpress (NJ, USA). Cycloheximide (CAS No. 66-81-9) was purchased from A.G. Scientific (CA, USA). Yuanhuadine (YD; purity >98.5%) was isolated from a CHCl_3_-soluble fraction of the flowers of *Daphne genkwa*, as described previously^22^. All chemicals were dissolved in DMSO for in vitro experiments.

Antibodies against C-terminal AXL (sc-1096), EGFR (sc-03), p-ERK (sc-7383), ERK (sc-94), MET (sc-10), β-actin (sc-47778) were obtained from Santa Cruz Biotechnology (Santa Cruz, CA, USA). p-AXL (#5724), p-EGFR (#2234), p-MET (#3077), p-Akt (#9271), Akt (#9272), p-p70S6 Kinase (#9205), p70S6 Kinase (#9202), p-SAPK/JNK (#9091), SAPK/JNK (#9252), and snail (#3879) were obtained from Cell Signaling Technology (Danvers, MA, USA).

### Cell culture

Human non-small lung cancer cells HCC827, HCC827-gef, PC9, PC9-gef cells were a kind gift of Dr. Jae Cheol Lee and Dr. Jin Kyung Rho (Asan Medical Center, Seoul, Korea). HCC827-gef and PC9-gef cells were subcultured in the presence of 1 µM gefitinib. Resistant cell line HCC827-osi was generated in vitro by culturing HCC827 cells with escalating doses (0.001–0.5 μM) of osimertinib. All the cells were maintained in RPMI 1640 media supplemented with 10% Fetal Bovine Serum (FBS) and 1% antibiotics-antimycotics (AA) (PSF; 100 units/mL penicillin G sodium, 100 μg/mL streptomycin, and 250 ng/mL amphotericin B).

### Cell viability assay

Cell viability was assayed by MTT method. Cells were seeded in 96-well plates. On the next day, cells were treated with indicated concentrations of gefitinib, osimertinib, or YD alone or in combination for 72 h. IC_50_ values were calculated via non-linear regression analysis using TableCurve 2D v5.01 (Systat Software Inc., San Jose, CA, USA). The combination effect was evaluated by the value of the combination index (CI) values which were calculated as follows: CI = D_1_/(D_*x*_)_1_+D_2_/(D_*x*_)_2_. D_1_ and D_2_ are the concentrations of the combined test compounds that achieve the expected effect, and (D_*x*_)_1_ and (D_*x*_)_2_ are the concentrations that achieve similar effects when the test compounds are used alone. The CI values were compared with the reference values reported by Chou^23^.

### Gene knockdown using siRNA transfection

siRNAs were purchased from Invitrogen. Negative control siRNA from the same company was used for control. After seeding, cells were transfected with 100 pmol siRNA duplex for 24 h using Lipofectamine RNAiMAX (Invitrogen, CA, USA) according to the manufacturer’s instructions. The coding strand for *AXL* was as follows: sense CCA GCA CCU GUG GUC AUC UUA CCU U and antisense AAG GUA AGA UGA CCA CAG GUG CUG G.

### Western blotting analysis

The cells were lysed in 2× sample loading buffer (250 mM Tris-HCl pH 6.8, 4% SDS, 10% glycerol, 0.006% bromophenol blue, 2% β-mercaptoethanol, 50 mM sodium fluoride, and 5 mM sodium orthovanadate). Tumor tissues were collected in RIPA buffer (Thermofisher, Rockford, IL, USA), and then further lysed with 2x laemmli sample buffer with 2% β-mercaptoethanol (Biorad). The collected samples were subjected to 6-12% SDS-PAGE gel and transferred onto PVDF membranes (Millipore, Bedford, MA, USA). The membranes were blocked with 5% BSA in Tris-buffered saline containing 0.1% Tween-20 (TBST) for 1 h at room temperature, and then incubated with primary antibodies in 2.5% BSA in TBST overnight at 4 °C on a shaker. The membranes were washed three times with TBST and incubated with the secondary antibodies (HRP) (Younginfrontier, Seoul, Korea) diluted in TBST for 2 h at room temperature. After washing with TBST, the membranes were exposed to enhanced chemiluminescence (ECL) solution (Intron, Daejon, Korea). The chemiluminescence signals were captured using LAS-4000 (Fuji Film Corp., Tokyo, Japan).

### Real-time PCR analysis

The total RNA of the cells was isolated with TRI reagent (Invitrogen, Grand Island, NY, USA). The isolated RNA (1 µg) was reverse-transcribed using ReverTra Ace qPCR RT Master Mix (TOYOBO, Osaka, Japan) according to the manufacturer’s instructions. Using synthesized cDNA, Real-time PCR was conducted using iQ^TM^ SYBR^®^ Green Supermix (Bio-Rad, Hercules, CA, USA), according to the manufacturer’s instructions. The comparative C_T_ method was used to determine the relative expression normalized by *β-actin*. The sequences of the primers are listed below.

*AXL*

(F) 5′-CGTAACCTCCACCTGGTCTC-3′;

(R) 5′-TCCCATCGTCTGACAGCA-3′

*GAS6*

(F) 5′-CATCAACAAGTATGGGTCTCCGT-3′;

(R) 5′-GTTCTCCTGGCTGCATTCGTTGA-3′

*β-actin*

(F) 5′-AGCACAATGAAGATCAAGAT-3′;

(R) 5′-TGTAACGCAACTAAGTCATA-3′

### Immunocytochemistry

The cells were grown on a confocal dish pre-coated with 0.2% gelatin. The cells were fixed with 4% paraformaldehyde (in PBS) for 15 min and were blocked in 1% BSA (in PBS containing 0.1% Triton X-100) for 30 min at room temperature. Cells were incubated with primary antibody (AXL, 1:50) at 4 °C overnight and further incubated with secondary antibody (anti-mouse Alexa 647, 1:250) for 2 h at room temperature. The nuclei were stained with DAPI (0.5 µg/ml). The images were detected using a confocal microscope (Leica, TCS SP8).

### Tumor xenograft study

Balb/c-nu mouse (male, 4-weeks-old; OrientBio, Seoul, Korea) were allowed one-week acclimation prior to the experiment. HCC827 (2 × 10^6^ cells), HCC827-gef (4 × 10^6^ cells), or HCC827-osi (4 × 10^6^ cells) cells were prepared in 100 µl PBS and mixed with the equal amount of Matrigel (Corning, Bedford, MA, USA) right before injecting subcutaneously into the flanks of the mice. When the tumor volume reached 50 mm^3^ (HCC827) and 100 mm^3^ (HCC827-Gef, HCC827-osi) on average, the mice were randomized into the vehicle control and treatment groups (*n* = 5). Drugs were mixed with vehicle (EtOH:Tween80:Saline solution 1:1:98). Each drug was administrated orally once a day and 6 times per week for 22 days (HCC827-gef, HCC827-osi) and 90 days (HCC827). The body weight and tumor size were measured every 3–7 days. The tumor size was measured using a digital slide caliper and volumes (mm^3^) were calculated as follows: (width × length × height) × π/6. The normalized tumor volume as follows: (TV_j,treated_/TV_i,control_), where TV_i_ is the initial tumor volume of first administration, and TV_j_ is the tumor volume of day j. Animals were sacrificed after the final drug administration and tumors were collected for ex vivo analysis.

### Patient-derived xenograft study

Patient-derived tumor specimens were collected at Yonsei University Severance Hospital. The study protocol was approved by the institutional review board of Severance Hospital (4-2013-0526), and all patients provided written informed consent. Tumors and paired peripheral blood samples were consecutively collected for PDX establishment and further genetic analysis. PDXs were created using 6–8-week-old female severe combined immunodeficient (NOG) and nude (nu/nu) mice (OrientBio, Seoul, Korea). The tumors and related PDXs were assigned Yonsei Human In Mouse (YHIM) identifiers that corresponded to the original patient-derived tumors. Tumor dimensions were measured twice a week with a digital caliper and tumor volume was calculated as follows: (length × width^2^)/2. Establishment of acquired gefitinib-resistant PDX tumors (YHIM-1009) and drug administration was performed in Yonsei Cancer Center and carried out as described previously^24^.

### Immunohistochemistry staining

The tumors after the end of xenograft experiment were excised, fixed in 4% paraformaldehyde (in PBS), and embedded in paraffin. The embedded specimens were sectioned carefully, serially deparaffinized, rehydrated, and subjected to antigen retrieval. Immunohistochemical analysis of the tumor tissues was carried out as described previously with the indicated antibodies^25^.

### Statistical analysis

The data are presented as the means ± SD for the minimum three independently performed experiments. The statistical significance (*P *<* 0.05, **P *<* 0.01, ***P *<* 0.005) were determined by ANOVA using Dunnett’s test. All statistical tests were two-sided.

## Results

### Differential sensitivity to osimertinib is observed in EGFR-mutant acquired gefitinib resistant NSCLC cells

**Fig. 1:**
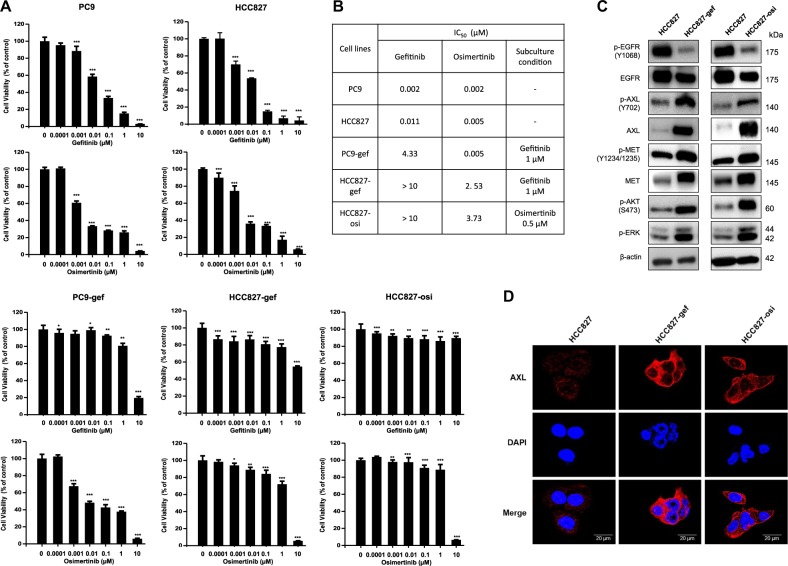
Overexpression of AXL is observed in EGFR-TKI resistant NSCLC cells. NSCLC cells (PC9, PC9-gef, HCC827, HCC827-gef, and HCC827-osi) were treated with gefitinib or osimertinib for 72 h. Cell viability was measured by MTT assay as described in Methods (**a**). IC_50_ values and subculture conditions were stated (**b**). The protein expressions of major RTKs, p-AKT, and p-ERK were determined by Western blotting (**c**). Cells were incubated on the confocal dish for 24 h, fixed, and stained with C-term AXL (red) and DAPI (blue) for visualization of cellular localization (**d**). Data are presented as the mean fold changes ± SD of three independent experiments. *P < 0.05, **P < 0.01, ***P < 0.005 by *t*-test

To investigate the molecular mechanism of EGFR-TKIs resistance, we primarily evaluated the effects of gefitinib and osimertinib, the first and third generation of EGFR-TKIs, respectively, on growth of EGFR-mutant NSCLC cell lines after 72 h of treatment (PC9, PC9-gef, HCC827, HCC827-gef, HCC827-osi) (Fig. 1a). While both HCC827 and PC9 cells harbor the EGFR Exon 19 deletion mutation, HCC827 has been reported to have higher MET expression while PC9 has higher EGFR and FGFR3 expression among NSCLC cell lines harboring mutant EGFR status^26^. Resistant cells were established through continuous exposure to gefitinib or osimertinib using a dose-escalation procedure, and finally they exhibited at least 100-fold greater IC_50_ than did the parental cells. We found that PC9 and HCC827 cells were initially very sensitive to both gefitinib and osimertinib (Fig. 1b). Interestingly, two gefitinib-resistant cells showed different sensitivity to osimertinib. Osimertinib effectively inhibited the growth of PC9-gef cells, but not HCC827-gef cells. Moreover, acquired osimertinib resistant cells developed from HCC827 also became intrinsically resistant to gefitinib. This suggests acquired resistant cells established from HCC827 cells develop EGFR-independent resistance mechanism.

### AXL is a major bypass pathway in osimertinib resistant cells

In order to investigate the mechanisms of acquired resistance of HCC827-gef and HCC827-osi cells, the expression levels of major receptor tyrosine kinases (RTKs) and major downstream targets were evaluated by Western blotting. The activation of phosphorylated Akt and ERK has been reported to drive resistance and growth in tumor cells^27,28^. In HCC827-gef and HCC827-osi cells, both AXL and MET were activated in compensating for EGFR inactivation. In addition, the levels of phosphorylated Akt and ERK were elevated compared with those of HCC827 cells (Fig. 1c). The expressions of AXL in HCC827, HCC827-gef and HCC827-osi cells were also evaluated by immunocytochemistry staining of C-term AXL (Fig. 1d). AXL was clearly overexpressed in the resistant cells and mainly localized in the cytosol.

**Fig. 2:**
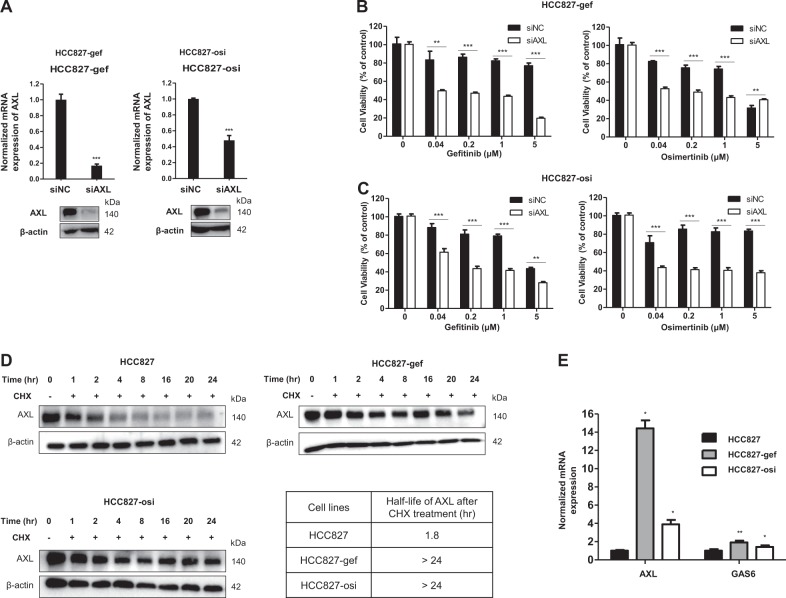
The total AXL protein degradation rate is extended in EGFR-TKI resistant cells. The efficiency of siAXL was confirmed using real-time PCR and Western blotting (**a**). The effects of gefitinib or osimertinib on the cell viability were evaluated after cells were transfected with siNC or siAXL compared with control (no transfection) in HCC827-gef cells (**b**) and HCC827-osi cells (**c**). Cycloheximide (CHX) was treated to stop the protein production and the protein expression of AXL (140 kda) was detected by Western blotting over the indicated time points. The expressions of AXL and β-actin were quantified by densitometry using ImageJ. AXL expressions were normalized to β-actin and further compared with 0 h expressions (**d**). mRNA expression of AXL and GAS6 were detected by real-time PCR using β-actin as a reference gene (**e**). Data are presented as the mean fold changes ± SD of three independent experiments. *P < 0.05, **P < 0.01, ***P < 0.005 by *t*-test

To examine the role of AXL in the resistant cells, the effects of AXL knockdown on the drug sensitivity of EGFR-TKIs in HCC827-gef and HCC827-osi cells were evaluated. The efficiency of siRNA was confirmed prior to the experiments (Fig. 2a). AXL inhibition significantly restored the sensitivity of gefitinib and osimertinib in both HCC827-gef and HCC827-osi cells (Fig. 2b, c). Although AXL was overexpressed in PC9-gef cells, knockdown of AXL was less effective in restoring the gefitinib sensitivity (Supplementary Fig. S1a–c). Together, these results suggest AXL is the major bypass pathway and plays a significant role in HCC827-gef and HCC827-osi cells which are resistant to both gefitinib and osimertinib.

### Overexpression of AXL is associated with extended protein degradation rate

To further assess the status of AXL in the resistant cells compared with the parent cells, we measured the AXL protein degradation rate using Western blotting analysis. The protein degradation rate of full-length AXL was measured after blocking new protein synthesis using cycloheximide (CHX). Both HCC827-gef and HCC827-osi cells displayed extended protein degradation rates of AXL than that of HCC827 parent cells (Fig. 2d). Western blotting bands of AXL expression was normalized by β-actin expression to quantify half-life. The half-life of AXL protein was calculated to be 1.8 h in HCC827 cells, while it was longer than 24 h in the resistant cells. A ligand of AXL, GAS6 binds and activates AXL receptor signaling thereby stimulating the cell proliferation^29^. We found that the gene copy number of AXL and its ligand genes, GAS6, were increased in the resistant cells (Fig. 2e). AXL was found to be overexpressed in PC9-gef cells, so we also compared the half-life of AXL in PC9 and PC9-gef cells. The overexpression of AXL was also correlated with extended degradation rate in PC9-gef cells (Supplementary Fig. S1d). These results indicated that resistant cells exhibit higher GAS6 and AXL expression and AXL overexpression is closely associated with extended protein degradation rates.

### YD inhibits cell proliferation and by AXL degradation in resistant cells

**Fig. 3:**
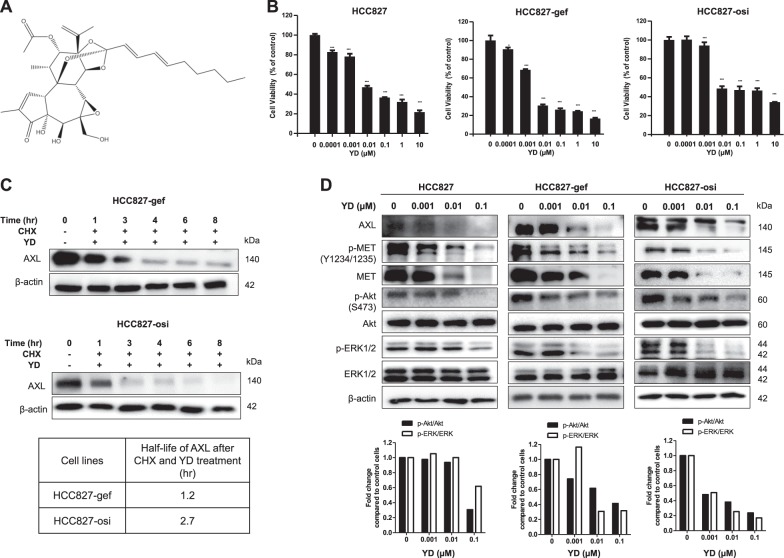
YD can regulate the degradation of AXL and effectively inhibit the growth of both EGFR-TKI sensitive and resistant cells. Chemical structure of yuanhuadine (YD) (**a**). Indicated cell lines were treated with YD for 72 h andviability was measured. (**b**). The protein expression of AXL (140 kda) was detected after co-treatmentof CHX and YD by Western blotting analysis over the indicated time points to test the effects of YD onthe protein degradation. The expressions of AXL and β-actin were quantified by densitometry usingImageJ. AXL expressions were normalized to β-actin and further compared with 0 h expression (**c**). Cells were treated with indicated concentrations of YD for 24 h (**d**). Data are presented as the mean foldchanges ± SD of three independent experiments. *P < 0.05, **P < 0.01, ***P < 0.005 by *t*-test

YD, derived from the flowers of *Daphne genkwa*, inhibits AXL expression by targeting protein degradation (Fig. 3a)^30^. To access the effects of AXL degradation on cell viability, we examined the effects of YD in HCC827, HCC827-gef, and HCC827-osi cells. The treatment of YD for 72 h reduced cell viability of HCC827, HCC827-gef, and HCC827-osi with IC_50_ value of 7.06 nM, 2.92 nM, and 9.2 nM, respectively (Fig. 3b). Moreover, YD treatment accelerated the degradation of AXL protein of resistant cells. The calculated half-life of AXL protein after YD treatment was 1.2 h in HCC827-gef cells and 2.7 h in HCC827-osi cells which became similar to that of parent cells (Fig. 3c). The effects of YD on the AXL signaling pathway were further examined in both sensitive and resistant cells. YD concentration-dependently inhibited full-length AXL and its downstream targets including phosphorylated Akt and ERK without affecting the total protein expression (Fig. 3d). Interestingly, YD suppressed the levels of phosphorylated MET and total MET expressions. Collectively, YD efficiently regulates AXL degradation, MET, and their downstream signaling in both EGFR-TKI-sensitive and EGFR-TKI-resistant cells.

### YD resensitizes HCC827-gef and HCC827-osi cells to EGFR-TKIs

**Fig. 4:**
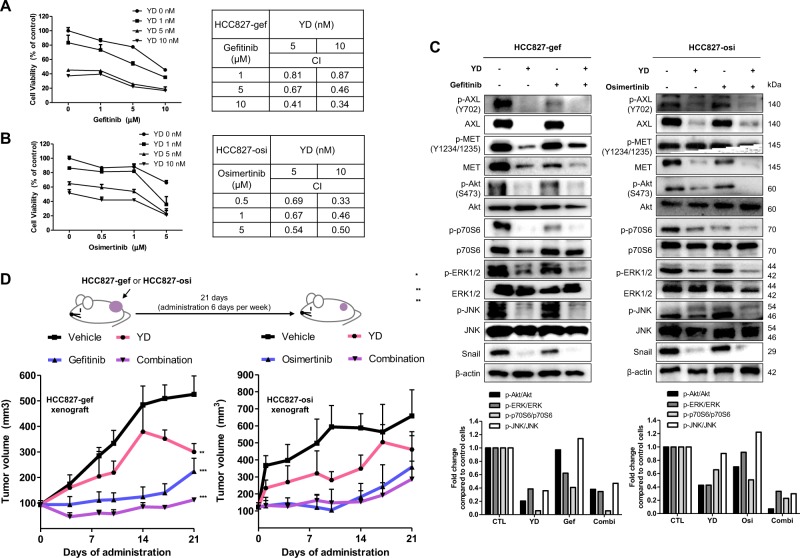
YD and EGFR-TKIs synergistically inhibit the growth of gefitinib and osimertinib cross-resistant cells in in vitro and in vivo. Cell viability was measured after combined treatment of YD and gefitinib or osimertinib for 72 h in HCC827-gef (**a**) and HCC827-osi (**b**), respectively. Based on the cell viability results, CI values were calculated to demonstrate the combination effects in each cell line. The effects of combination treatment compared with single treatment were accessed using western blot. Cells were treated with YD (10 nM) and/or gefitinib (1 μM) in HCC827-gef cells, and YD (10 nM) and/or osimertinib (500 nM) in HCC827-osi cells (**c**). HCC827-gef cells were subcutaneously implanted into the flanks of Balb/c-nude mice (*n* = 5). Mice were orally administered with compounds 6 times per week for 21 days and doses indicated are 1 mg/kg for YD, 10 mg/kg for gefitinib. HCC827-osi cells were subcutaneously implanted into the flanks of Balb/c-nude mice (*n* = 5). Mice were orally administered with compounds 6 times per week for 21 days and doses indicated are 0.5 mg/kg for YD, 5 mg/kg for osimertinib (**d**). Data are presented as the mean fold changes ± SD of three independent experiments. *P < 0.05, **P < 0.01, ***P < 0.005 by *t*-test

AXL kinase inhibitors are reported to resensitize EGFR-TKIs resistant cells to EGFR-TKIs^31^. We further evaluated whether AXL degrader, YD, could resensitize resistant cells to gefitinib and osimertinib. Treatment of YD with gefitinib or osimertinib for 72 h inhibited cell growth more than any single drug treatment (Fig. 4a left and B left). These synergistic effects were evaluated by combination index (CI) analysis using the Chou-Talalay method, which is based on the median-effect equation and also provide algorithms for computer simulation^23,32^ (CI < 1: synergism, CI = 1: additive; CI > 1: antagonism). The formula can be applied for any dose-effect analysis and even suitable for small-scale experiments. Various doses of compounds were tested to accurately determine the effects of drug combinations. The calculated CI values were smaller than 1, which indicates synergism (Fig. 4a right and b right).

Treatment with either gefitinib or osimertinib alone did not have significant effects on the expression of AXL, MET, and downstream signaling in resistant cells. On the other hand, the combination of YD and EGFR-TKIs strongly inhibited the expressions of p-Akt, p-ERK, p-p70S6, and p-JNK which regulate cell proliferation and survival (Fig. 4c). Moreover, YD and combined treatment inhibited snail expression, which is one of the major EMT target genes regulated by AXL signaling^33^. In search of potential application of YD in a combination therapy, we also investigated the combined effects of YD and other kinase inhibitors such as PHA-665752 (c-MET inhibitor), SP600125 (JNK inhibitor), and LY294002 (PI3K inhibitor) which were showed limited efficacy as a single agent in the resistant cells. Combined treatment with YD resulted in significant suppression of cell viability and showed synergism with all of three kinase inhibitors (Supplementary Fig. S2). These results indicate targeting AXL degradation by YD can resensitize resistant cells not only to EGFR-TKIs, but also potentially to c-MET inhibitor, JNK inhibitor, or PI3K inhibitor.

### Combined administration of YD with EGFR-TKIs synergistically inhibits tumor growth in xenograft model bearing HCC827-gef and HCC827-osi cells

To further confirm the synergistic activity of YD and EGFR-TKIs using in vivo model, we examined their efficacy in a xenograft model engrafted with HCC827-gef and HCC827-osi cells. The drugs were orally administered once a day, 6 days per week, for 3 weeks. Resistant cells developed in vitro were implanted in the right flank of nude mice. After one week, mice were randomly assigned to each group. Consistent with in vitro data, the combination administration effectively inhibited the tumor group in both HCC827-gef and HCC827-osi xenograft tumor model (Fig. 4d). YD as a single treatment group resulted in tumor-regression after 14 days in HCC827-gef tumor model and 17 days in HCC827-osi tumor model. Collectively, the combination of YD and EGFR-TKIs shows potential for both inhibitions of in vitro cell proliferation and in vivo tumor growth via AXL degradation.

### Combined administration of YD with gefitinib synergistically inhibits tumor growth of gefitinib-resistant PDX model

**Fig. 5:**
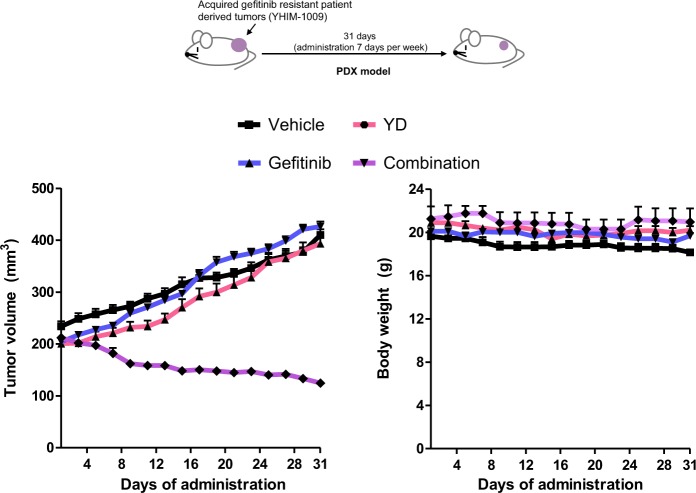
YD and EGFR-TKIs synergistically inhibit the growth in acquired gefitinib-resistant PDX models. When tumor volumes reached 200 to 250 mm3, mice engrafted with YHIM-1009 tumors were segregated into appropriate treatment groups (three to five mice per group). Mice were orally administered with compounds every day for 31 days and doses indicated are 1 mg/kg for YD, 10 mg/kg for gefitinib (**a**). *P < 0.05, **P < 0.01, ***P < 0.005 by *t*-test

In order to assess the combination effects of YD and gefitinib in more clinically relevant setting, we compared activity of single agent administration and combination administration in acquired gefitinib-resistant patient derived xenograft (PDX) model. The third mouse generation of YHIM-1009 (EGFR 19del mutation/ PIK3CA E542K) tumor model, an acquired-gefitinib resistant tumor, was used for drug efficacy testing. The previous study demonstrated that YHIM-1009 did not show response to both gefitinib (25 mg/kg) and osimertinib (6.25 mg/kg) administration^24^. When tumor volumes reached 200 to 250 mm^3^, animals were administrated with vehicle, YD (1 mg/kg), gefitinib (10 mg/kg), or YD and gefitinib every day for 31 days. Combined administration of YD and gefitinib notably inhibited the tumor growth derived from acquired gefitinib-resistant patient tumor more efficiently than a single treatment (Fig. 5a). Bodyweight was measured to monitor the toxicity, and there was no significant change in bodyweight for all groups. Unlike the results of HCC827-gef xenograft model, YD single administration resulted in only slight inhibition of tumor growth. These results indicate that YD potentially more useful for combination therapy than as a single agent.

### Combined administration of YD with gefitinib delays the emergence of resistance in long-term xenograft model bearing HCC827 cells

**Fig. 6:**
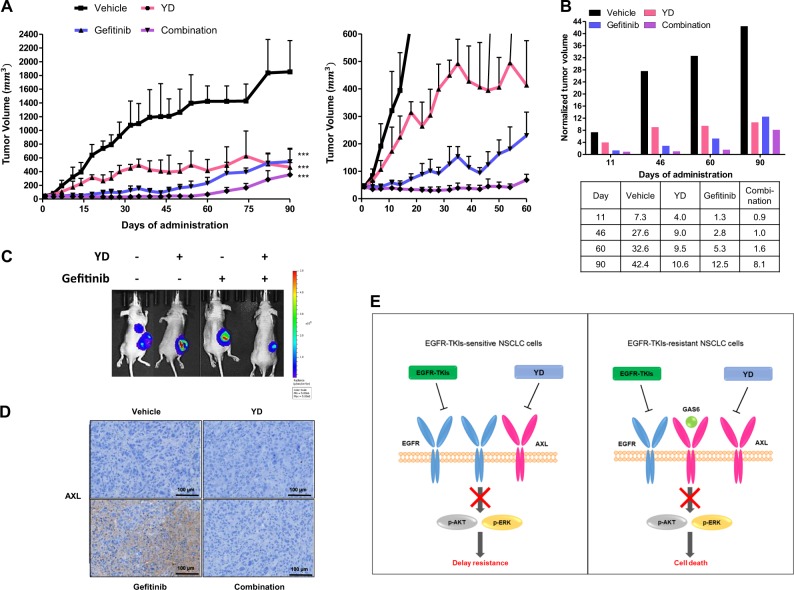
The combined administration of YD and gefitinib can delay the emergence of resistance and inhibit AXL overexpression in a long-term in vivo xenograft model. HCC827-Luc cells were subcutaneously implanted into the flanks of Balb/c-nude mice (five mice per group). Drugs were orally administered 6 times per week for 90 days and doses indicated are 0.5 mg/kg for YD, 10 mg/kg for gefitinib (**a**). The graph represents tumor volumes of indicated days which were normalized to the initial tumor volume for comparison (**b**). Bioluminescence images of mice at the final day before sacrifice were measured and mice were injected with Firefly D-luciferin before imaging (**c**). Immunohistochemical analysis of AXL was performed in tumor tissue (**d**). The schematic diagram illustrating the significance of combined YD with EGFR-TKI treatment in NSCLC (**e**). *P < 0.05, **P < 0.01, ***P < 0.005 by *t*-test

Because AXL is regarded as an acquired resistance mechanism to EGFR-TKIs, we hypothesized that AXL inhibition with EGFR-TKIs from the beginning of treatment would prevent the emergence of acquired resistance, enhance the therapeutic efficacy, and extend drug response time. Therefore, we performed a long-term (90 days) xenograft experiment using a xenograft model implanted with EGFR-TKI-sensitive HCC827 cells (Fig. 6a left). Not every vehicle group survived throughout the long-term experiment due to the tumor burden, so remaining mice were still used to record tumor size and body weights. We observed no change in body weights in combination group and gefitinib group, while the body weight was only slightly reduced in YD group (data not shown). Combination of YD and gefitinib both almost completely prevented the tumor growth for 60 days, while the tumors of the gefitinib administration group exceeded the initial size and started to grow rapidly after 35 days of drug administration (Fig. 6a right). The tumor volumes on day 11, 46, 60, and 90 were normalized to the initial tumor volume before the administration to quantify the effects (Fig. 6b). Tumors in the combination group later started to grow after day 60, but their final volumes were still smaller than those of the YD group or gefitinib group (Fig. 6c). Although tumor size in the vehicle-administered group was much larger, AXL expression was undetected in immunohistochemistry staining. These data confirm that AXL increases in response to the long-term administration of gefitinib regardless of tumor size (Fig. 6d). Combined treatment with YD and gefitinib was not resulted in the AXL expression in the tumors. However, the tumor sizes of the combination treatment group also increased at later points in time, suggesting that tumors may have acquired resistance by additional mechanisms. Further studies are needed to investigate the mechanism involved with resistance to the combination treatment of YD and gefitinib. Collectively, these results suggest that using a combined treatment as a first-line therapy may delay the emergence of acquired-resistance and as a second-line therapy may overcome resistance (Fig. 6e).

## Discussion

Although the mutation and activation of EGFR are recognized as important oncogenic drivers in NSCLC patients, targeting EGFR using currently existing EGFR-TKIs has shown limited success due to the emergence of acquired resistance^34^. Osimertinib, a third-generation TKIs, has been developed to treat mutant EGFR or/and T790M-positive NSCLC patients and also has been reported to have superior efficacy as first-line treatment^35^. A recent study showed the activation of AXL as an adaptive response to osimertinib, and SPRY4 as a potential negative regulator of AXL signaling in lung cancer^36^. However, little has been studied about the roles of overexpressed AXL in acquired resistance mechanisms against osimertinib. Our study report that extended degradation rate of AXL is pivotal in the development of resistance to gefitinib, osimertinib; thus, we investigated therapeutic approaches using drug combination to overcome resistance by targeting AXL degradation.

Targeting protein degradation may have an advantage over kinase inhibition in the regulation of cancer cell growth; thus the efficacy of drugs with proteolysis-targeting chimera (PROTAC) technology is under investigation^37,38^. Several studies with EGFR, HER2, and c-MET inhibitors have confirmed that targeted protein degradation may have an advantage because it yields more sustained loss of functions and prevents kinome re-wiring^37^. Along these lines, we utilized the natural antitumor compound YD to target AXL degradation which exhibits anti-proliferative activities against various human lung cancer cells by regulating AXL, SerpinB2, NNMT, and BMP4^22,39–42^. YD exhibits nanomolar IC_50_ in NSCLC cell lines which is much lower than currently existing AXL kinase inhibitors based on their reported IC_50_ value^31^.

We previously found that YD cleaves AXL independent of kinase, metalloprotease, proteasome and lysosome activities^30^. Treatment of YD can release N-terminal into the culture medium and sequentially generate intracellular domain thereby degrading the full-length AXL. When N-terminal of AXL is cleaved, it may work as a decoy receptor to inhibit the binding of GAS6 and AXL receptor. For example, an engineered AXL decoy receptor, which antagonizes the GAS6/AXL system by capturing GAS6, can inhibit cancer cells and tumor growth without toxicity^43^. Therefore, N-terminal fragment generated by AXL may prevent binding of GAS6 and AXL receptor thereby enhancing the AXL blockade activity. YD inhibited AXL, as well as MET protein expression which resulted in more sustained inhibition of downstream signaling pathway. Previous study also reported that tumor suppressor OPCML inactivates AXL-dependent oncogenic signaling in ovarian cancer and also prevent transactivation of other RTKs such as EGFR and cMET^44^.

It is well reported that AXL inhibition using kinase inhibitors can sensitize or synergistically work with several drugs such as antimitotic drugs, erlotinib, and imatinib^8,17,19^. In addition, several studies suggested that there are crosstalks between AXL and other RTKs; (1) AXL clusters together with other cell surface RTKs in the mesenchymal cells which leads to crosstalk-amplified signaling network and a limited response to single TKIs treatment^45^. (2) When head and neck and esophageal squamous cell carcinomas become resistant to PI3Kα inhibition, AXL dimerizes with EGFR and activates EGFR/PKC/mTOR pathway^46^. (3) AXL not only forms a complex with EGFR but also may promote nuclear translocation of the EGFR^47,48^. (4) MGCD265, which is a multi-targeted TKIs targeting MET, AXL, and PDGFR, completely inhibited the growth in only tumors with high MET gene copy gains, and combination of MGCD265 and erlotinib effectively inhibited the tumor growth in most of triple breast cancer cells and tumors^49^. (5) AXL kinase inhibitor (R428) suppressed EMT-induced metastasis and combined treatment enhanced the effects of lapatinib or trastuzumab in HER2-overexpressed breast cancer PDX model^50^. These studies support combination treatment ought to be superior than single drug treatment.

In this study, we confirmed that AXL protein degradation with EGFR-TKIs synergistically suppresses the growth of EGFR mutant cells and tumors. The effects of combined administration were very significant in PDX model. The tumor model used in PDX harbors EGFR mutation, as well as PI3KA mutation, which may explain results of inefficient YD single administration group. Nonetheless, the combined administration showed very potent tumor growth inhibition. As previously reported, tumor tissues obtained from gefitinib-administrated group in a long-term xenograft study with HCC827 cells showed significantly higher AXL expression from the immunohistochemistry results^17^. Although tumor size in the vehicle-administered group was much bigger in long-term xenograft, the AXL expression was negligible. These findings suggest that AXL is upregulated in response to the long-term administration of gefitinib, regardless of tumor size, and affect sensitivity of tumors to EGFR-TKIs. When administrated in combination of YD and gefitinib, AXL was not detected in the tumors from the immunohistochemistry results. These results indicate that YD completely suppressed the AXL activation and was able to delay the tumor regrowth.

In conclusion, we have elucidated AXL as a driver of cross-resistance to gefitinib and osimertinib and demonstrated the efficacy of combining YD with gefitinib and/or osimertinib in overcoming EGFR-TKI resistance in cells, resistant tumor xenograft and PDX model, and in delaying the emergence of acquired resistance. These results suggest combination use of YD and EGFR-TKIs may represent a potential therapeutic strategy for NSCLC patients.

## Supplementary information

Supplementary Figure 1-2

## Data Availability

Please contact authors for data request.
