# Supplementary material for: AXL degradation in combination with EGFR-TKI can delay and overcome acquired resistance in human non-small cell lung cancer cells
Source: Cell Death Dis. 2019 May 1;10(5):361. doi: 10.1038/s41419-019-1601-6 (PMC6494839; doi:10.1038/s41419-019-1601-6)
Supplement: Supplementary file 1 — Supplementary Figure 1-2 [file 41419_2019_1601_MOESM1_ESM.pdf]

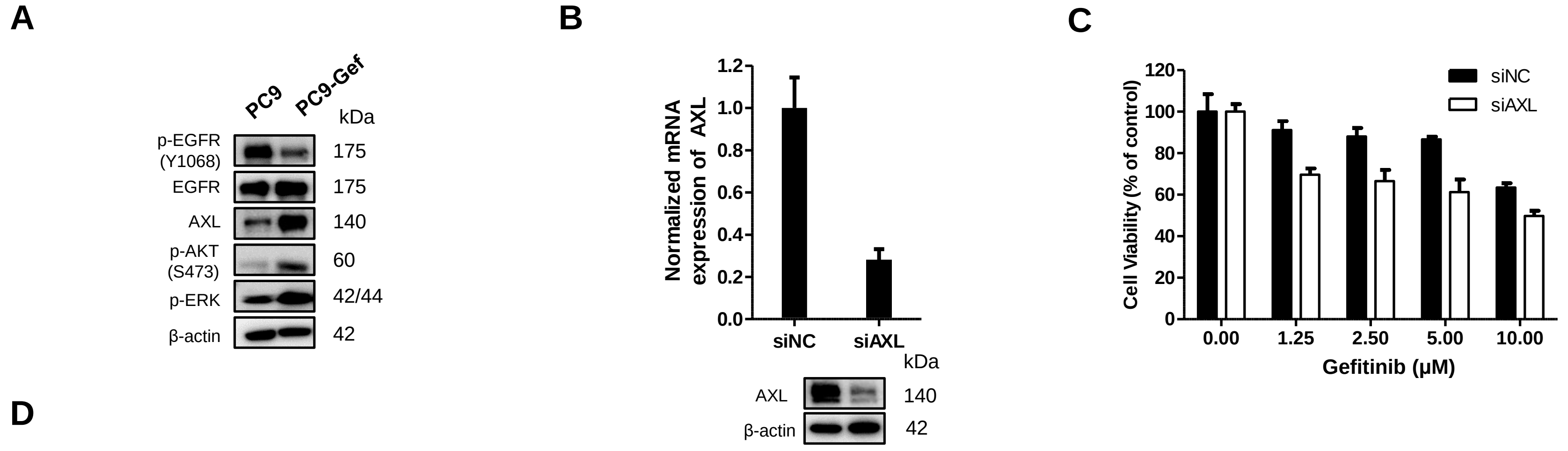

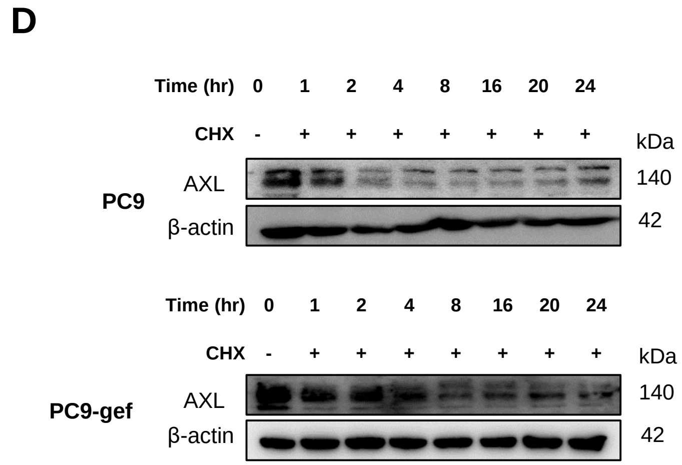

Cell lines	Half-life of AXL after CHX treatment (hr)
PC9	2.1
PC9-gef	16.7

Supplementary Figure. S1

A

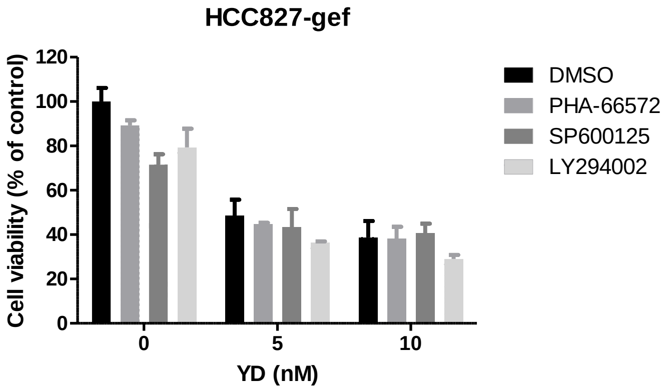

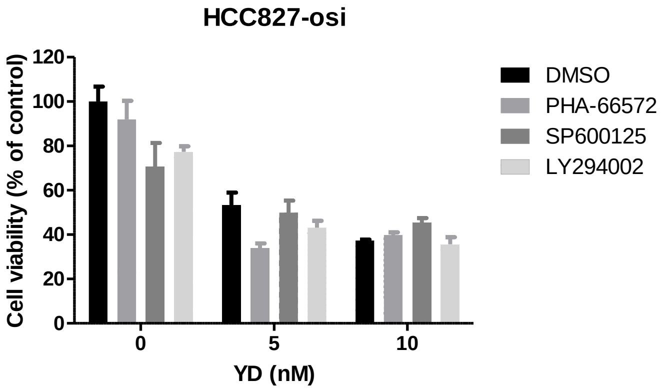

B

HCC827-gef

	YD 5 nM		YD 10 nM	
PHA-665752 (μM)	CI	Descrirption	CI	Descrirption
0.16	0.190	Strong synergism	0.335	Synergism
0.8	0.237	Strong synergism	0.237	Strong synergism
4	0.594	Synergism	0.548	Synergism
20	0.195	Strong synergism	0.192	Strong synergism

	YD 5 nM		YD 10 nM	
SP600125 (μM)	CI	Descrirption	CI	Descrirption
0.16	0.355	Synergism	0.355	Synergism
0.8	0.322	Synergism	0.322	Synergism
4	0.238	Strong synergism	0.238	Strong synergism
20	0.141	Strong synergism	0.141	Strong synergism

	YD 5 nM		YD 10 nM	
LY294002 (μM)	CI	Description	CI	Descrirption
0.16	0.306	Synergism	0.360	Synergism
0.8	0.209	Strong synergism	0.340	Synergism
4	0.191	Strong synergism	0.215	Strong synergism
20	0.214	Strong synergism	0.181	Strong synergism

HCC827-osi

	YD 5 nM		YD 10 nM	
PHA-665752 (μM)	CI	Descrirption	CI	Descrirption
0.16	0.140	Strong synergism	0.126	Synergism
0.8	0.064	Very strong synergism	0.060	Very strong synergism
4	0.179	Strong synergism	0.201	Strong synergism
20	0.044	Very strong synergism	0.045	Very strong synergism

	YD 5 nM		YD 10 nM	
SP600125 (μM)	CI	Descrirption	CI	Descrirption
0.16	0.135	Synergism	0.186	Strong synergism
0.8	0.196	Synergism	0.101	Synergism
4	0.300	Strong synergism	0.304	Synergism
20	0.737	Strong synergism	0.644	Synergism

	YD 5 nM		YD 10 nM	
LY294002 (μM)	CI	Descrirption	CI	Descrirption
0.16	0.213	Synergism	0.436	Synergism
0.8	0.263	Strong synergism	0.512	Synergism
4	0.396	Strong synergism	0.342	Strong synergism
20	0.952	Strong synergism	0.881	Strong synergism

PHA-665752: c-MET inhibitor
SP600125: JNK inhibitor
LY294002: PI3K inhibitor

Supplementary Figure. S2
